# True hermaphroditism with sex cord tumor with annular tubules (SCTAT): a rare case report and review of the literature

**DOI:** 10.1186/s12905-022-02137-7

**Published:** 2022-12-27

**Authors:** Suhua Shi, Ming Tang, Wuan Li, Haixing Wu, Yinhua Liu, Yonghong Luo, Huafeng Ding

**Affiliations:** 1 Department of Obstetrics and Gynecology, The First Affiliated Hospital of Wannan Medical College, No. 2 Zheshan West Road, Wuhu, 241000 China; 2 Department of Pathology, The First Affiliated Hospital of Wannan Medical College, Wuhu, China

**Keywords:** True hermaphroditism, Sex cord tumour with annular tubules, Surgery, Hormonal replacement

## Abstract

**Background:**

True hermaphroditism is a rare condition. It is defined as the presence of both testicular and ovarian tissues in the same individual. Sex cord tumour with annular tubules (SCTAT) is a rare stromal tumour of the sex cord that occurs mostly in the ovaries.

**Case presentation:**

A 16-year-old girl presented to the gynaecology department with primary amenorrhea. Gynaecological examination revealed an enlarged clitoris that looked like a small penis. The chromosome karyotype was chimaera. The postoperative pathology confirmed true hermaphroditism with SCTAT. The patient underwent hormonal replacement after an operation and had no evidence of recurrence for 6 months.

**Conclusion:**

Cases of true hermaphroditism with SCTAT are extremely rare conditions. Surgery and hormonal replacement are important for improving the prognosis of such patients.

## Background

True hermaphroditism is a rare condition and is defined as the presence of two types of gonadal tissue (ovarian and testicular) in one patient, regardless of the patient’s karyotype [1]. The incidence rate is 1 in 100,000 live births [2]. Sex cord tumour with annular tubules (SCTAT) is a rare stromal tumour of the sex cord that occurs mostly in the ovaries but occasionally in the fallopian tubes and testicles, and approximately 15%-20% of SCTAT cases tend to be clinically malignant [3, 4]. Approximately one-third of patients have Peutz-Jeghers syndrome (PJS). There is no standard treatment for SCTAT due to the rarity of cases.

In this study, we present a case of a 16-year-old patient with true hermaphroditism combined with SCTAT treated in our hospital. The clinical manifestations, histological morphology, diagnosis and treatment are discussed along with a literature search. As a routine procedure, written informed consent was obtained from the patient to collect clinical and pathological data for publication.

## Case presentation

**Fig. 1:**
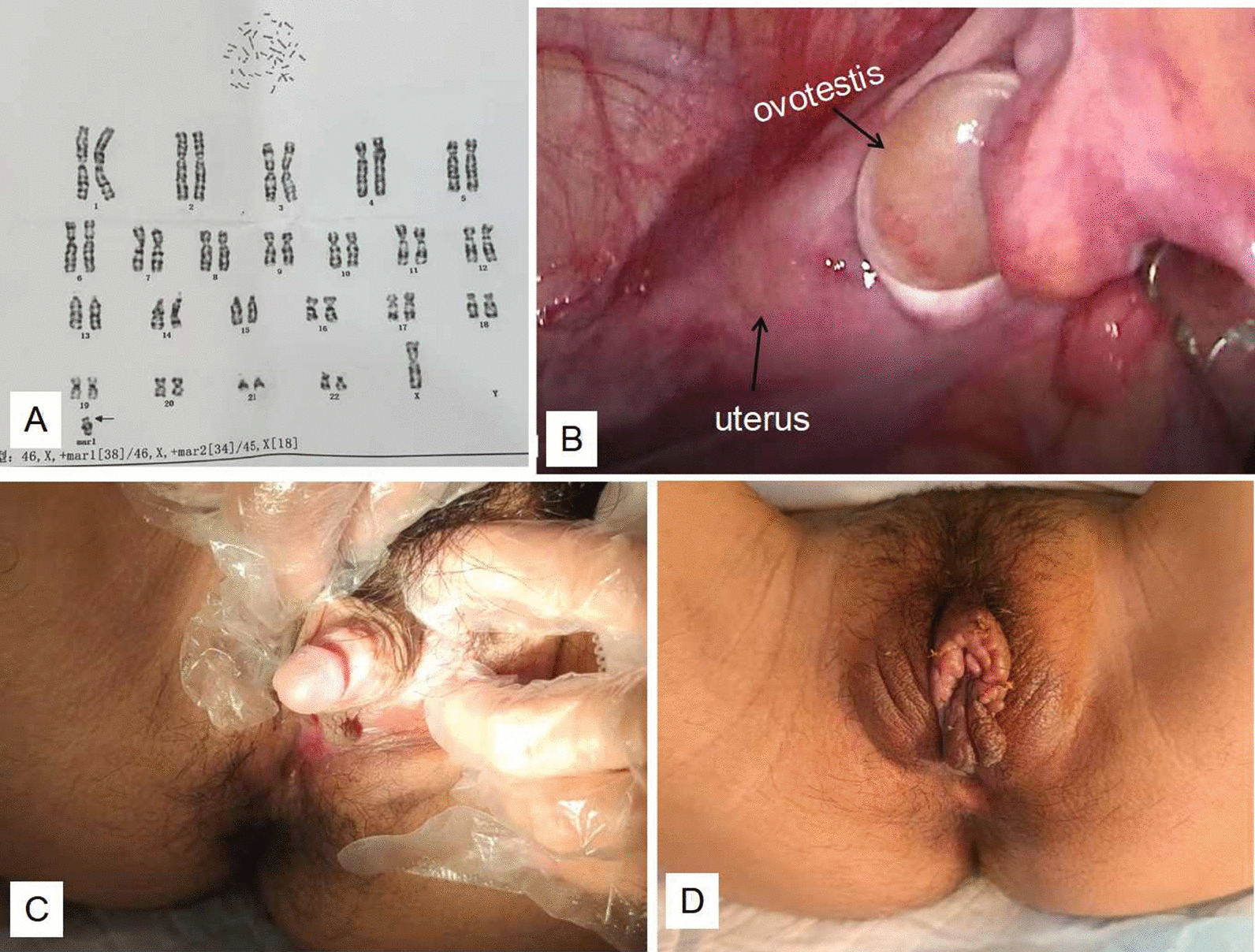
**A** Chromosomal analysis showed: 46,X,+mar1[38]/46,X,+mar2[34]/45,X[18]. **B** Laparoscopic exploration revealed a rudimentary uterus, immature fallopian tubes (right), and ovotestis (right). **C** Preoperative gynaecological examination revealed an enlarged clitoris that looked like a small penis. **D** The shape of vulva recovered 2 weeks later after the clitoroplasty

A 16-year-old girl presenting with primary amenorrhea was admitted to the gynaecology department on August 12, 2021. Physical examination showed that the patient had short stature (height: 151 cm, weight: 46 kg, BMI: 20.17 kg/m^2^), poor breast development and hypertrichosis. Gynaecological examination revealed that pubic hair was distributed in a male pattern, and clitoral hypertrophy gave the appearance of a small penis with a clitoral body length of approximately 4 cm and a diameter of 1.5 cm (Fig. 1). The positions of the urethral orifice and vaginal orifice were normal. The depth of the vagina was 6 cm. Colour Doppler ultrasonography showed that the uterine body was approximately 34 × 25 × 35 mm, the cervix was approximately 20 × 15 mm, the endometrium was not clear, and the size of the ovaries was 25 × 12 mm (left) and 25 × 17 mm (right). Ultrasonography of both the kidneys and adrenal glands showed no abnormalities. On laboratory assessment, testosterone was 8.4 nmol/l, oestradiol was 14 pg/ml, luteinizing hormone was 36.33 mIU/ml, and follicle stimulating hormone was 70.79 mIU/ml. Pituitary prolactin, adrenocorticotropic hormone, cortisol, and thyroid function tests were normal. A chromosomal analysis was performed, and the result showed that there was 46,X,+mar1[38]/46,X,+mar2[34]/45,X[18]. To identify the subtelomeric microdeletion of the chromosome region, a chromosomal microarray analysis (CMA) was performed, and the results showed arr(X)x1,Yp11.32q11.223(1-21924023)x0-1. According to the results of clinical and laboratory examinations, the patient was diagnosed with true hermaphroditism before the operation.

**Fig. 2:**
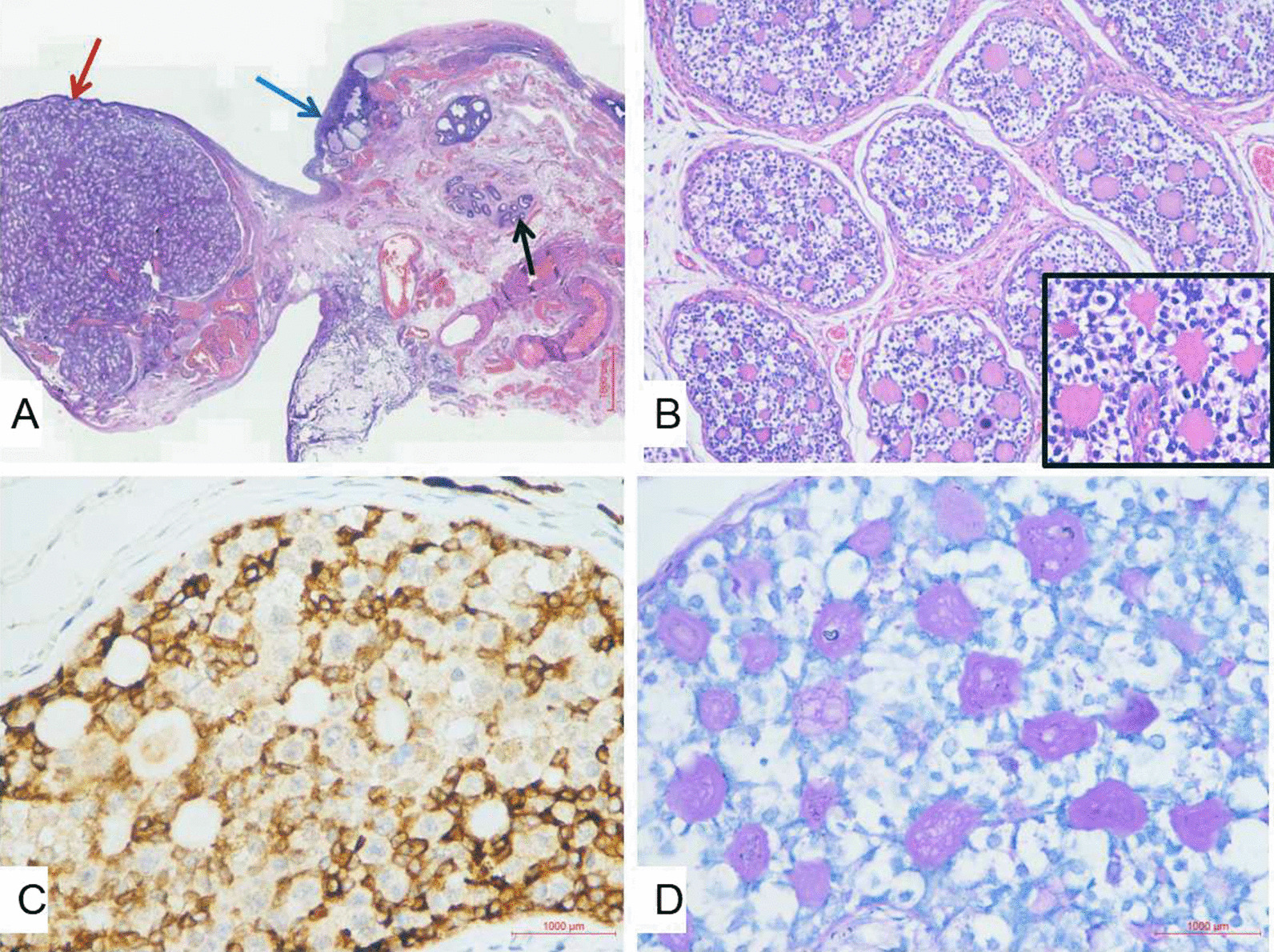
**A** Hematoxylin eosin stain, × 30: Features of vary (blue arrow), testis (red arrow), and epididymis (black arrow) in the structure removed. Pathological and immunohistochemical features of SCTAT (**B**–**D**). Hematoxylin eosin stain, × 100: Cell block section with annular tubules with eosinophilic hyaline cores (Inset: H&E × 400). **C** Immumohistochemical stain, × 400: positive a-inhibin staining in the cytoplasm. **D** Special staining, × 400: PAS staining positive in annular tubules

True hermaphroditism cases must be treated as either a man or a woman based on age, sex gonads, external genitalia and orientation. Since the patient was socially and psychologically female in every aspect, after full communication with the patient and her parents, plastic surgery of the external genitalia and single-hole laparoscopic bilateral salpingo-oophorectomy was performed on the patient. Laparoscopic exploration revealed a rudimentary uterus, bilateral immature fallopian tubes, and bilateral ovotestis with sizes of 1.5 cm × 2.5 cm (right) and 1.5 cm × 2 cm (left) (Fig. 1). Clitoroplasty with preservation of the dorsal vascular nerve bundle of the clitoris was performed. Histopathological examination revealed features of the fallopian tube, ovary, testis and epididymis in the structure removed. These morphological characteristics were consistent with true hermaphroditism. Tumour cells with simple annular tubules with eosinophilic hyaline cores could be seen under a microscope. Immunohistochemical results showed that tumour cells were AE1/AE3 (−), EMA (−), inhibin-A (+), AFP (partially weakly +), calretinin (−), CD30 (−), PLAP (partially +), and WT1 (partially +), and the Ki-67 positivity rate was 30% while PAS staining was positive in the annular tubules (Fig. 2). Therefore, the diagnosis of SCTAT was also confirmed. After 2 weeks of follow-up, the patient was satisfied with the plastic restoration of the vulva (Fig. 1). To maintain the female characteristics, hormone replacement therapy with fenbutone (oestradiol 1 mg, oestradiol 1 mg and didroxyprogesterone 10 mg) was administered after the operation. During the 6-month follow-up period, she had no evidence of recurrence. Her menstruation was regular, but the amount of menstruation was small.

## Discussion and conclusions

The aetiology and pathogenesis of true hermaphroditism is not completely clear. It is considered to be related to factors such as sex chromosome abnormalities, abnormal gonadal development and related endocrine disorders in the process of embryonic development [5]. In recent years, studies have found that many genes, such as the sex-determining region (SRY) of the Y chromosome, anti-Mullerian hormone (AMH) gene, Wilm’s tumour gene-1 (WT-1), steroidogenic factor-1 (SF-1), dose-sensitive sex reversal congenital adrenal dysplasia gene-1 (DAX-1), and sex-determining region Y-box protein 9 (Sox-9), are involved in the differentiation of the gonads with sexual potential. Abnormal changes in any of these genes can affect the normal differentiation and development of the gonads, leading to the occurrence of hermaphroditism. In addition, a large number of epidemiological studies have shown that the impact of environmental pollution (exogenous-related hormones) on reproductive system malformations, especially exposure to oestrogen and progesterone substances during early and middle pregnancy, can also affect the normal differentiation of foetal gonads and increase the risk of abnormal sexual development [6–8]. True hermaphroditism is extremely rare. The karyotypes of patients with true hermaphroditism can be 46, XX, 46, XY or chimaeric bodies. According to the location of the two gonads, hermaphroditism can be classified into the following: (1) bilateral—an ovotestis is present on both sides, accounting for approximately 20% of cases; (2) unilateral—an ovotestis is present on one side, and a testis or ovary is present on the other side, accounting for approximately 40% of cases; and (3) lateral—an ovary is present on one side, and a testis is present on the other side, accounting for approximately 40% of cases [9]. Patients with hermaphroditism usually present malformations of the internal and external genitalia. The male genital phenotype may manifest as a small penis, hypospadias, or cryptorchidism. The female phenotype may present hypertrophy of the clitoris, which ranges from a significantly enlarged clitoris to a penis-like clitoris. Most of these patients also have uterine hypoplasia and absent or aplastic vaginas. The treatment of true amphoterism mainly involves sex selection and reconstruction of the internal and external genitalia, including surgery and hormone replacement therapy. It is generally accepted that poorly developed testes or ovotestes with no clear boundary between the ovarian tissue and testicular tissue should be removed early, along with close follow-up to avoid the occurrence of malignant tumours [10]. This case presents a 16-year-old girl with primary amenorrhea. The chromosome karyotype was chimaera. Pathological examination revealed the existence of two gonads in the patient, which is typical true hermaphroditism.

Sex cord tumours with annular tubules (SCTAT) are rare and distinctive, accounting for < 1% of all sex cord stromal tumours [11]. SCTAT has morphologic features intermediate to those of granulosa cell tumours and Sertoli cell tumours [12]. SCTAT was first described as a distinctive tumour by Scully in 1970 [13]. There are two clinical forms: the hereditary form, occurring as bilateral, multifocal tumours in patients with PJS, and the sporadic form, occurring as a solitary neoplasm in patients without evidence of the syndrome. The size of the tumour can vary from tiny masses only observed under a microscope to bulky masses of more than 10 cm. It is very difficult to diagnose SCTAT before surgery, and pathology and immunohistochemistry have important reference values. Microscopically, round, fused nests of tumour cells can be seen, forming a single or composite closed circular tubule with eosinophilic hyaline substance at the core. Immunohistochemically, the tumour cells are positive for alpha-inhibin, vimentin, and calretinin and negative for epithelial membrane antigen (EMA). The diagnosis of SCTAT in the patient without symptoms in the present case was confirmed by histopathological examination. And it was maybe not really related to the hermaphroditism. Due to the scarcity of cases, there is no established standard treatment regimen for SCTAT, and the initial treatment is surgery [14]. Qian et al. [15] reported that SCTAT had a recurrence rate of 46.2%. After surgery or adjuvant treatment, most recurrences were well controlled; the 1- and 5-year progression-free survival (PFS) rates were 92% and 67%, respectively, and the median PFS was 97.8%. This research has shown that SCTAT has potential malignant behaviour, and its recurrence rate is high, but the prognosis is good. Therefore, fertility-preserving surgery is feasible. Although the patient in this case had undergone bilateral adnexectomy, close follow-up and regular review of B ultrasound were still required. During the 6-month follow-up period, the patient had no evidence of recurrence.

In conclusion, in this study, we report one of the rarest conditions: a patient with true hermaphroditism and bilateral ovotestes with SCTAT. It is extremely rare that all of these conditions would exist in one patient. Surgery and hormonal replacement are important for improving the prognosis of such patients. To the best of our knowledge, this is the first report of its kind in the literature.

## Data Availability

The datasets used during the current study are available from the corresponding author on reasonable request. Department of Obstetrics and Gynecology, the First Affiliated Hospital of Wannan Medical College, Wuhu, China.

## Abbreviations

SCTAT: Sex cord tumor with annular tubules
PJS: Peutz-Jeghers syndrome
CMA: Chromosomal microarray analysis
AMH: Anti Mullerian hormone
SRY: Sex determining region
WT-1: Wilm's tumor gene-1
SF-1: Steroidogenic factor-1
DAX-1: Dose sensitive sex reversal congenital adrenal dysplasia gene-1
Sox-9: Sex determining region Y-box protein 9
